# Этиопатогенетические особенности костного метаболизма пациентов с сахарным диабетом, осложненным диабетической нейроостеоартропатией

**DOI:** 10.14341/probl13362

**Published:** 2024-09-15

**Authors:** М. В. Ярославцева, О. Н. Бондаренко, Я. А. Эль-Тарави, С. Т. Магеррамова, Е. А. Пигарова, И. Н. Ульянова, Г. Р. Галстян

**Affiliations:** Национальный медицинский исследовательский центр эндокринологии; Национальный медицинский исследовательский центр эндокринологии; Национальный медицинский исследовательский центр эндокринологии; Национальный медицинский исследовательский центр эндокринологии; Национальный медицинский исследовательский центр эндокринологии; Национальный медицинский исследовательский центр эндокринологии; Национальный медицинский исследовательский центр эндокринологии

**Keywords:** сахарный диабет, медиакальциноз артерий нижних конечностей, метаболиты витамина D, костный метаболизм, фосфорно-кальциевые нарушения, диабетическая нейроостеоартропатия, стопа Шарко, отек костного мозга, минеральная плотность кости

## Abstract

Дистальная диабетическая нейропатия является одним из наиболее распространенных осложнений сахарного диабета (СД) и ассоциируется с медиакальцинозом артерий нижних конечностей, значительным снижением минеральной плотности костной ткани стоп и высокой частотой сердечно-сосудистых заболеваний (ССЗ). Медиакальциноз является индикатором тяжести и/или продолжительности заболевания, поскольку тесно связан с осложнениями диабета, в особенности с автономной нейропатией. Наличие медиакальциноза является важным маркером будущих сердечно-сосудистых нарушений у пациентов с СД. Изменения фосфорного-кальциевого обмена встречаются у пациентов с диабетической нейроостеоартропатией или стопой Шарко, когда наблюдается локальный остеопороз стоп и в 90% случаев происходит обызвествление сосудов нижних конечностей, а также изменение уровня витамина D и его метаболитов. Длительность течения СД и состояние компенсации значительно влияют на процесс минерально-костных нарушений. В данном обзоре обсуждаются особенности метаболизма витамина D, важность своевременной диагностики фосфорно-кальциевых нарушений и особенности терапии данной категории больных. Уделяется внимание своевременной диагностике острой стадии стопы Шарко на основании оценки отека костного мозга при проведении МРТ и возможности уменьшения срока иммобилизации пораженной конечности за счет его регресса.

## Abstract (en)

Diabetic neuropathy is one of the most common diabetes mellitus complications associated with mediocalcinosis of the lower extremities, a significant decrease in feet bone mineral density, and a high incidence of cardiovascular disease. In most cases, calcium-phosphorus metabolism changes occur in patients with diabetic neuroarthropathy, or Charcot foot, when we can observe feet local osteoporosis, which in 90% of cases associated with a vessel’s calcification of the lower extremities in the majority of diabetes population. A large number of studies presented literature have demonstrated that patients with Charcot foot can have accelerated bone metabolism and increased bone resorption. Patients with Charcot foot often have crucial abnormalities in the calcium-phosphorus parameters, bone metabolism, and levels of vitamin D and its metabolites. In addition, the duration of diabetes mellitus, the degree of its compensation widely affects the development of its micro- and macrovascular complications, which could also accelerate the development of mineral and bone disorders in these types of patients. Multifactorial pathogenesis of these disorders complicates the management of patients with a long and complicated course of diabetes mellitus. This review discusses the peculiarities of vitamin D metabolism, the importance of timely diagnosis in phosphorus-calcium disorders, and the specifics of therapy in these patients. Special attention is paid to the timely diagnosis of the Charcot’s foots acute stage based on the bone marrow edema by MRI evaluation and the possibility of reducing the immobilization period.

## ВВЕДЕНИЕ

Диабетическая нейроостеоартропатия (ДНОАП), или стопа Шарко — тяжелое инвалидизирующее состояние, ассоциированное с сахарным диабетом (СД) и диабетическая нейропатия (ДН) [1], проявляющееся стойкой выраженной деформацией стопы с нарушением ее биомеханических свойств и высоким риском формирования обширных язв, что может приводить к развитию гнойно-некротического процесса в мягких тканях стопы и остеомиелиту, повышая риск ампутаций. Пациенты с неудовлетворительным контролем гликемии в течение длительного периода заболевания, сопутствующими хроническими микрососудистыми осложнениями, такими как хроническая болезнь почек и ДНОАП, имеют значимо более высокий риск последовательных структурных и гемодинамических нарушений, клинические последствия которых ассоциируются с высоким риском сердечно-сосудистых заболеваний (ССЗ) и смерти [2][3]. Сведения о частоте ДНОАП чрезвычайно вариабельны, от 0,15% всех пациентов с диабетом до 29% в популяции пациентов с ДН [4]. Противоречивость результатов обусловлена различной трактовкой изменений костной ткани, а также использованием неравнозначных по информативности методов диагностики. Различные исследования по распространенности остеоартропатии выявили высокую частоту ее развития у пациентов с длительностью диабета более 12 лет независимо от возраста, пола и типа диабета. У большинства пациентов процесс односторонний, тогда как двустороннее поражение встречается в 9–25% случаев [5].

Метаболические и структурные изменения в костной ткани при ДНОАП сопряжены с медиакальцинозом (МК) артерий нижних конечностей вследствие ДН, одного из наиболее распространенных осложнений СД. У пациентов со стопой Шарко наблюдается локальный остеопороз стоп, и в 90% случаев происходит обызвествление сосудов стоп и голеней (рис. 1 А, Б) [3] . Выявляемый рентгенологически МК артерий нижних конечностей нередко ошибочно принимают за облитерирующий атеросклероз, что приводит к неверной тактике лечения. Обызвествление стенок артерий может обнаруживаться и у лиц без нарушений углеводного обмена, причем с возрастом частота МК возрастает (от 5% у молодых до 37% у пожилых). При СД МК выявляется в среднем в 3 раза чаще, чем у лиц с нормальным углеводным обменом [6]. По данным ряда исследований, распространенность МК была выявлена у 17–42% пациентов с СД 2 типа (СД2) [7], у 27–40% пациентов с прогрессирующей ХБП [8].

**Figure:**
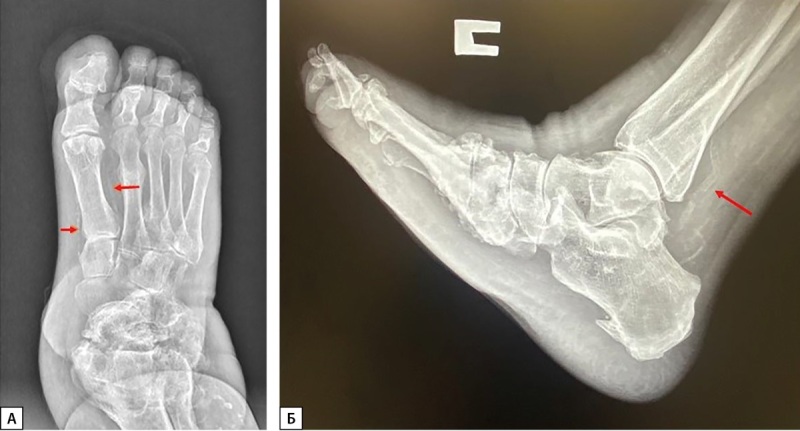
Рисунок 1. А. Рентгенограмма левой стопы пациента с ДНОАП в прямой проекции. (стрелками обозначен медиакальциноз артерий стопы).Б. Рентгенограмма правой стопы пациента с ДНОАП в боковой проекции (стрелками обозначен медиакальциноз артерий стопы).

МК является индикатором тяжести и/или длительности заболевания, поскольку он тесно связан с осложнениями диабета, особенно с автономной нейропатией и хронической болезнью почек (ХБП). Данная категория больных находится в группе риска развития минерально-костных нарушений (МКН), которые в свою очередь могут усугублять течение ДНОАП, усиливая резорбцию костной ткани и эктопическую кальцификацию, клиническим следствием которой являются сердечно-сосудистые события и повышение смертности таких пациентов. Особенности метаболизма витамина D в патогенезе МКН при ДНОАП остаются малоизученными [9][12].

Известно, что пациенты с СД имеют повышенный риск переломов по сравнению с лицами без диабета. В 2021 г. Rabe et al. провели ретроспективный анализ большой группы пациентов с ДНОАП, указывающий на повышенный риск переломов и остеопороза у больных со стопой Шарко в сравнении с больными с СД без Шарко [13]. Исследования минеральной плотности кости (МПК), измеренной при помощи рентгеноденситометрии у пациентов с ДНОАП, показали снижение МПК пораженной стопы по сравнению с контрлатеральной конечностью. Однако данных о снижении МПК в позвоночнике и бедре нет, и это позволяет предположить, что костная патология у данной категории больных носит локальный характер [14][15].

Патогенез фосфорно-кальциевых нарушений у пациентов с СД малоизучен. На процесс ремоделирования костной ткани у пациентов с СД могут влиять такие факторы, как длительность СД, контроль заболевания, наличие хронических осложнений СД, в том числе ХБП, проведение заместительной почечной терапии (ЗПТ) или трансплантация солидных органов и получение иммуносупрессивной терапии. В литературе также обсуждается связь дефицита некоторых витаминов с ДНОАП [16][17]. В данной статье будет рассмотрено влияние перечисленных факторов на развитие стопы Шарко и их связь с дефицитом витамина D и его метаболизма.

Многофакторность патогенеза ДНОАП затрудняет ведение пациентов с длительным и осложненным течением СД. В данном обзоре обсуждаются этиопатогенетические особенности костного метаболизма у пациентов с СД, осложненным ДНОАП, важность своевременной диагностики фосфорно-кальциевых нарушений и особенности терапии у данной категории больных. Уделяется внимание своевременной диагностике острой стадии стопы Шарко на основании оценки отека костного мозга (ОКМ) при проведении МРТ и возможности уменьшения срока иммобилизации пораженной конечности за счет его регресса.

## ЭТИОЛОГИЯ

Стопа Шарко обычно проявляется в виде асептического воспаления и прогрессирующей дегенерации костной ткани, что может приводить к спонтанным переломам и необратимым костным деформациям, что по сути проявляется разобщением процессов синтеза и резорбции костной ткани [1]. Существует гипотеза, что развитие ДНОАП запускается факторами, увеличивающими кровоток в конечностях при наличии у пациента изначальной предрасположенности к данному заболеванию вследствие наличия у него ДН. Такими факторами могут быть: легкая травма, предшествующая язва стопы и даже реваскуляризация. Однако, несмотря на доказанный факт развития остеоартропатии лишь при наличии ДН, прогнозировать ее развитие практически невозможно, так как она возникает далеко не у всех больных даже с выраженной нейропатией.

Согласно данным ряда исследований, МПК у больных СД выявляются в 30–67% случаев на уровне остеопении, а остеопороз — в 7–47% [17][18]. Сегодня существует множество исследований МПК у больных СД, показывающих, что риск переломов возрастает с увеличением продолжительности диабета и наличием его поздних осложнений. Так, нарушение функции почек приводит к снижению МПК, а диабетическая нейропатия, снижение остроты зрения и цереброваскулярные заболевания — к высокому риску падений и травмам нижних конечностей. Поскольку неудовлетворительный контроль гликемии ассоциирован с высоким риском переломов, большое внимание уделяется влиянию лекарственной терапии диабета. Исследование Meier et al. показало, что противодиабетические препараты из группы тиазолидиндионов, такие как пиоглитазон, увеличивают риск переломов у женщин с СД2 за счет снижения МПК, риск у мужчин менее очевиден [19]. В некоторых исследованиях метформин был связан со снижением риска переломов, а препараты сульфонилмочевины не влияли на МПК или частоту переломов. Ряд исследований инкретиномиметиков (ситаглиптин, лираглутид) продемонстрировал протективное действие на МПК [20], в экспериментальных исследованиях назначение аГПП-1 и иДПП-4 ассоциировалось с усилением костеобразования и снижением костной резорбции [21]. В исследованиях продемонстрирована статистически значимая связь применения канаглифлозина с высокой частотой переломов [22][23]. Напротив, применение других препаратов из группы ингибиторов НГЛТ-2 (эмпаглифлозин, дапаглифлозин) не влияет на риск развития переломов. Учитывая, что неудовлетворительный контроль СД снижает МПК и увеличивает риск переломов, необходима дальнейшая исследовательская работа над этими группами препаратов, чтобы уточнить их влияние на МПК.

## ПАТОГЕНЕЗ

Изучение патогенеза стопы Шарко эволюционировало от нейротравматической и нейроваскулярной теории до концепции остеокласто-остеобластного дисбаланса, вызывающего резорбцию кости, деминерализацию и остеопению. Данная теория основана на взаимодействии локальных молекулярно-биологических механизмов, в том числе остеопротегерина (osteoprotegerin — OPG) и лиганда рецептора-активатора ядерного фактора каппа-В (RANKL), являющихся членами семейств лигандов и рецепторов фактора некроза опухолей (tumor necrosis factor — TNF). Исследования последних лет свидетельствуют о том, что они играют ключевую роль в формировании, дифференцировке и активности клеток костной ткани [24]. RANKL является ключевым фактором активации остеокластов (ОК). Связываясь со специфическим рецептором ОК, который обозначается как рецептор-активатор ядерного фактора каппа-В (RANK), RANKL стимулирует резорбирующую активность этих клеток. Полагают, что характер ремоделирования костной ткани во многом определяется соотношением продукции OPG и RANKL. OPG, связывая RANKL, предотвращает активирующее влияние последнего на RANK ОК, что снижает как остеокластогенез, так и активность ОК. Ряд исследований показал, что ОК пациентов с острой стадией стопы Шарко проявляли повышенную резорбтивную активность под воздействием RANKL [25], но опубликованы данные, показавшие, что повышенное соотношение RANKL/OPG присутствует только в острой стадии заболевания и не является постоянно повышенным у людей со стопой Шарко.

Система OPG/RANKL играет важную роль как в процессе кальцификации артерий, так и развитии локальной остеопении у больных СД [26]. Несмотря на большое количество исследований, посвященных системе RANK/RANK-L/OPG, а также существование моноклонального антитела к RANK-L Деносумаба, неясно, можно ли использовать остеопротегерин в качестве потенциальной мишени для разработки новых препаратов терапии МКН.

## ОСОБЕННОСТИ КОСТНОГО МЕТАБОЛИЗМА У БОЛЬНЫХ ХБП

Длительное течение СД с неудовлетворительным контролем заболевания закономерно ведет к развитию поздних осложнений, в том числе к снижению функции почек. Почки являются ключевым органом, участвующим в фосфорно-кальциевом обмене, а прогрессирование ХБП ведет к дисбалансу процессов костного метаболизма и развитию МКН. МКН при ХБП ассоциированы с аномальным ремоделированием костной ткани, почечной остеодистрофией и внескелетной кальцификацией [27]. Последнее приводит к избыточной кальцификации сосудов и клапанов сердца и увеличению смертности от сердечно-сосудистых заболеваний, как на додиализных стадиях ХБП [28], так и у пациентов на гемодиализе [29].

Остеопения и остеопороз встречаются у пациентов с ХБП в 2–3 раза чаще, чем в общей популяции [30–32]. Известно, что риск переломов достоверно увеличивается с возрастом, тяжестью ХБП и продолжительностью диализа у пациентов с ХБП 5Д [33–35]. При этом установлено, что повышенный риск смертности при переломе шейки бедра значительно выше у пациентов со сниженной функцией почек [36]. При ХБП уровни витамина D и всех его метаболитов находятся под влиянием множества патогенетических процессов: снижается активность почечных ферментов — участников метаболизма витамина D в силу уменьшения количества функционирующей почечной ткани; изменяется катаболизм витамина D вследствие высоких уровней фактора роста фибробластов-23 и паратиреоидного гормона (ПТГ), имеющих дискордантный эффект на активность 24-гидроксилазы; происходит нарушение его всасывания в кишечнике, а также значительные его потери с мочой при выраженной протеинурии [37]. Сложностью в поддержании адекватного уровня фосфорно-кальциевого обмена также является низкая комплаентность пациентов, которым показано лечение препаратами витамина D. При этом оптимальный уровень витамина D, обеспечивающий защиту от переломов, все еще требует уточнения.

Учитывая повышенный риск переломов у пациентов с СД [38], рабочая группа Международного фонда остеопороза (International Osteoporosis Foundation — IOF), крупнейшая в мире неправительственная организация, занимающаяся профилактикой, диагностикой и лечением остеопороза и связанных с ним заболеваний опорно-двигательного аппарата, предложила использовать в качестве порогового значения для диагностики остеопороза у пациентов с СД -2,0 SD по Т-критерию вместо -2,5 SD. Такое изменение алгоритмов диагностики остеопороза у пациентов с СД позволит выявлять снижение МПК на более ранних стадиях и назначать своевременное лечение данной категории больных [39].

Сегодня вопрос относительно степени изменения метаболитов витамина D и маркеров костного метаболизма при стопе Шарко на разных ее стадиях все еще остается открытым. Отдельно хотелось бы обсудить особенности изменения метаболитов витамина D в сыворотке крови у пациентов с длительным течением СД и наличием множественных его осложнений. Имеющиеся данные указывают на биохимическую противоречивость связи уровня витамина D и его метаболитов с СД и его осложнениями [40]. Luo et al. предположили, что это противоречие может быть связано с тем, что некоторые метаболиты витамина D могут быть значительно снижены на фоне целевых значений общего витамина D. По данным метаанализа, удалось доказать, что низкие уровни 25(OH)D3 и 1,25(OH)2D3 играют значительную роль в развитии диабетической ретинопатии [41]. Butler et al. установили, что у пациентов с СД2 также наблюдались более низкие уровни всех метаболитов витамина D, за исключением 3-epi-25(OH)D3 [42]. Стоит отметить, что исследований, посвященных изучению метаболитов витамина D у пациентов ДНОАП, в литературе найдено не было. Это диктует необходимость проведения таких работ и установления роли витамина D и всех его метаболитов в развитии стопы Шарко.

При критическом снижении функции почек и терминальной стадии ХБП инициируется заместительная почечная терапия. МПК у пациентов на гемодиализе значительно ниже, чем в популяции [43]. В дополнение к этому, согласно ряду исследований, распространенность фосфорно-кальциевых нарушений среди пациентов, находящихся на диализе, достоверно выше, чем у пациентов без диализа [44][45]. Изменения уровней ПТГ, кальция и фосфора в сыворотке крови ассоциированы с повышенной смертностью пациентов на ЗПТ [46][47]. Учитывая, что более чем у половины пациентов на ЗПТ имеются отклонения от нормы уровня ПТГ [48], критически важно строго контролировать параметры минерального обмена у данной группы пациентов и своевременно его корректировать. Кроме того, у пациентов, перенесших аллотрансплантацию почки, может развиваться особый фенотип нарушений минерального обмена [49]. Пока достоверно неясно, какой механизм развития подобных нарушений у пациентов доминирующий: усугубление имевшихся на фоне СД фосфорно-кальциевых изменений, использование влияющих на МПК глюкокортикостероидов в классических схемах иммуносупрессивной терапии или остеодистрофия и дисфункция диаметра трансплантата. У пациентов на ЗПТ проблема кальцификации сосудов и смерти от сердечно-сосудистых заболеваний стоит более остро [50].

## СВЯЗЬ КОСТНОГО МЕТАБОЛИЗМА С КОНЦЕНТРАЦИЕЙ ВИТАМИНА В12 И ФОЛИЕВОЙ КИСЛОТЫ

Ряд исследований указывает на наличие ассоциации гомоцистеина, витамина В12 и фолиевой кислоты с состоянием МПК, остеопорозом и развитием переломов у пациентов с СД и стопой Шарко [51][52]. В двух метаанализах подтверждается связь между высокой концентрацией гомоцистеина и риском переломов [53][54], а в исследовании Brattström et al. подтверждается, что такие показатели гомоцистеина ассоциированы с дефицитом витамина В12 и фолиевой кислоты [55]. Таким образом, дефицит витамина В12 и фолиевой кислоты могут быть ассоциированы с повышенным риском переломов у пациентов с СД. Кроме того, низкие концентрации витамина В12 коррелируют с низкими концентрациями остеокальцина в сыворотке крови. Однако остается неясным, уменьшает ли дополнительный прием витаминов группы В риск развития переломов. Кроме того, в литературе не было найдено исследований, посвященных изучению содержания витамина В12, фолиевой кислоты, гомоцистеина у больных со стопой Шарко. Широко известно, что витамин В12 необходим для правильного функционирования периферической и центральной нервных систем, обеспечивает передачу нервных импульсов. Недостаток витамина В12 (менее 400 пмоль/л) развивается у пациентов, получающих метформин (препарат первой линии, применяемый для лечения СД2), уже через 3–6 месяцев после начала терапии, что вызывает демиелинизацию и поражение нервных волокон с явлениями нейропатии. К данной группе риска также относятся пациенты после бариатрических вмешательств, у которых из-за нарушения всасывания высока вероятность развития дефицита витамина В12. Периферическая нейропатия увеличивает риск развития синдрома диабетической стопы, ДНОАП. Следовательно, своевременное лечение полинейропатии может привести к снижению риска развития ДНОАП. Существуют исследования, результаты которых показывают уменьшение симптомов полинейропатии у пациентов, длительное время дополнительно получающих витамин В12. Отмечается улучшение качества жизни, снижение болевого синдрома, однако в клинической практике назначение препаратов витамина В12 широкого применения не получило.

## ДИАГНОСТИКА

С учетом высокого процента ампутаций нижних конечностей у больных СД, последующей инвалидизации и послеампутационной летальности данной категории пациентов, особую актуальность приобретает проблема наиболее ранней диагностики стопы Шарко. Рентгенография стоп при ДНОАП может характеризоваться неизмененной картиной, поэтому на этом этапе у таких пациентов важно своевременное проведение МРТ стоп или трехфазной сцинтиграфии костей с целью выявления отека костного мозга (ОКМ), повышенной резорбции кости в пораженной стопе и локального снижения МПК. Основные сложности возникают при диагностике острой стадии стопы Шарко, когда при наличии типичной клинической картины рентгенографические изменения отсутствуют. В таких случаях необходимо назначение дополнительных методов исследования. Предложенная Е.А. Shantelau и G. Grutzner в 2014 г. классификация ДНОАП на основании клинической картины и результатов МРТ описывает обязательные и возможные качественные признаки поражения костной ткани на разных стадиях ДНОАП и не отражает количественную оценку этих изменений [56].

Преимуществом МРТ перед рентгенографией стопы является ее способность визуализировать мягкие ткани и ОКМ, что позволяет диагностировать ДНОАП уже на этапе ОКМ, внутрикостных кист и микропереломов, а также проводить дифференциальную диагностику между остеомиелитом и стопой Шарко. Внедрение количественной оценки ОКМ на МРТ позволит более точно оценить активность патологического процесса в костях и мягких тканях, а также уменьшить длительность иммобилизации пораженной конечности (за счет регресса ОКМ), что в свою очередь приведет к снижению частоты ампутаций.

МРТ является наилучшим методом визуализации мониторинга активности ДНОАП. До тех пор, пока на МРТ визуализируется ОКМ, необходимо продолжать лечебные мероприятия, в том числе разгрузку конечности при помощи индивидуальной разгрузочной повязки (ИРП), о чем подробнее будет сказано ниже. После значительного уменьшения или полного исчезновения ОКМ гипсовую повязку можно снять и рекомендовать пациенту специальную ортопедическую обувь и стельки.

Наличие столь эффективного метода визуализации активности ДНОАП диктует необходимость в разработке специальной клинической шкалы на основе данных МРТ стоп. Такая шкала может быть направлена как на диагностику острой стадии процесса, так и оценку эффективности терапии, и переход активности процесса в стадию ремиссии на основании выраженности ОКМ. Попытки ввести подобные шкалы в клиническую практику уже были [57][58]. Однако цели данных исследований были преимущественно направлены на изучение диагностики острой стадии ДНОАП, а их масштаб не позволяет пока в полной мере оценить эффективность и безопасность данной методики. Вопрос проведения крупных рандомизированных клинических испытаний является сегодня наиболее актуальным, и ответ на него позволит увеличить эффективность диагностики и лечения пациентов со стопой Шарко и улучшить их качество жизни.

## ЛЕЧЕНИЕ

На данный момент единственными эффективными методами терапии ДНОАП и предотвращением прогрессирования деформации поврежденной конечности являются иммобилизация и разгрузка пораженного сустава при помощи ИРП. Стандартом иммобилизации считается несъемная индивидуальная разгрузочная повязка (ИРП) из полимерных материалов Тotal Contact Cast. Иммобилизация конечности продолжается до исчезновения отека, гиперемии, снижения температуры кожных покровов пораженной стопы и рентгенологически или с помощью МРТ подтвержденной консолидации костных отломков. В большинстве случаев длительность лечения — не менее 4–6 месяцев, иногда достигает 1 года. Учитывая, что терапевтический подход в группе пациентов со стопой Шарко ограничен, а единственным значимым методом профилактики является поддержание гликемических показателей в пределах индивидуальных целевых норм, существует необходимость проведения новых исследований, которые позволят в будущем расширить спектр лечения. При наличии выраженной деформации стопы проводится хирургическое лечение — реконструктивное (с применением внешней или внутренней фиксации) и резекция пролабирующих/выступающих фрагментов костей стопы с последующей иммобилизацией и динамическим наблюдением.

Применяемые в различное время лекарственные препараты, влияющие на метаболизм костной ткани, для лечения стопы Шарко не доказали своей клинической эффективности и не нашли применения в рутинной практике. Инъекции бисфосфанатов, обладающие антирезорбтивным эффектом, применялись у пациентов с острой стадией ДНОАП и вызывали значительное снижение температуры кожи над пораженными участками стоп. Выявлялось также снижение маркеров резорбции костной ткани по сравнению с группой плацебо, однако применение бисфосфонатов ассоциировалось с развитием большего количества их побочных эффектов. Бисфосфонаты не сокращали время иммобилизации пораженной конечности в индивидуальной разгрузочной повязке. Более того, не доступны никакие данные относительно их долгосрочных эффектов. Применяемый в клинической практике назальный спрей с кальцитонином, обогащенным кальцием и влияющий непосредственно на систему RANKL/OPG у больных СД со стопой Шарко, продемонстрировал снижение маркеров костной резорбции через 3 месяца применения в сравнении с группой, получавшей только препараты кальция [59]. В своем исследовании Busch-Westbroek et al. применяли моноклональное антитело RANKL у больных с СД и ДНОАП. Однократную дозу антител RANKL (деносумаб) подкожно вводили 11 пациентам, на фоне чего отмечалось снижение сроков консолидации переломов пораженной конечности и сроков иммобиллизации в сравнении с контрольной группой на 70 дней [60]. Исследований влияния деносумаба на МПКТ и возникновение рисков переломов у больных СД сегодня в литературе нет. В 2019 г. Rastogi et al. провели аналогичное исследование, посвященное терипаратиду (рекомбинантный паратиреоидный гормон человека), препарату для лечения остеопороза, повышающему плотность и прочность костной ткани. Препарат вводился 10 пациентам с хронической стадией стопы Шарко, продемонстрировав увеличение скорости ремоделирования костной ткани и увеличение МПК [61]. Других подобных исследований у больных с СД и стопой Шарко не проводилось.

## ЗАКЛЮЧЕНИЕ

Представленный обзор литературы отражает многофакторность патогенеза поражения костной ткани у больных СД (ХБП, длительный прием иммуносупрессивной терапии, включающей глюкокортикоиды, периферическая полинейропатия тяжелой степени, ВГПТ, дефицит витаминов и их метаболитов), что определяет трудности коррекции и особенности течения заболевания. Требуется дальнейшее изучение выявленных особенностей метаболизма витамина D у пациентов с ДНОАП. Необходимы поиск и разработка препаратов, направленных на регенерацию костного матрикса у больных с ДНОАП и ХБП. А своевременная диагностика и лечение острой стадии ДНОАП позволят снизить риски инвалидизации у данной категории больных.

## ДОПОЛНИТЕЛЬНАЯ ИНФОРМАЦИЯ

Источники финансирования. Исследование выполнено за счет гранта Российского научного фонда №19-15-00243, https://rscf.ru/project/19-15-00243/

Конфликт интересов. Авторы декларируют отсутствие явных и потенциальных конфликтов интересов, связанных с содержанием настоящей статьи.

Участие авторов. Все авторы одобрили финальную версию статьи перед публикацией, выразили согласие нести ответственность за все аспекты работы, подразумевающую надлежащее изучение и решение вопросов, связанных с точностью или добросовестностью любой части работы.
